# Feasibility of dopamine as a vector-valued feedback signal in the basal ganglia

**DOI:** 10.1073/pnas.2221994120

**Published:** 2023-08-01

**Authors:** Emil Wärnberg, Arvind Kumar

**Affiliations:** ^a^Department of Neuroscience, Karolinska Institutet, 171 77 Stockholm, Sweden; ^b^Division of Computational Science and Technology, School of Electrical Engineering and Computer Science, KTH Royal Institute of Technology, 114 28 Stockholm, Sweden

**Keywords:** basal ganglia, feedback alignment, learning, dopamine

## Abstract

It is well established that midbrain dopaminergic neurons support reinforcement learning (RL) in the basal ganglia by transmitting a reward prediction error (RPE) to the striatum. In particular, different computational models and experiments have shown that a striatum-wide RPE signal can support RL over a small discrete set of actions (e.g., no/no-go, choose left/right). However, there is accumulating evidence that the basal ganglia functions not as a selector between predefined actions but rather as a dynamical system with graded, continuous outputs. To reconcile this view with RL, there is a need to explain how dopamine could support learning of continuous outputs, rather than discrete action values. Inspired by the recent observations that besides RPE, the firing rates of midbrain dopaminergic neurons correlate with motor and cognitive variables, we propose a model in which dopamine signal in the striatum carries a vector-valued error feedback signal (a loss gradient) instead of a homogeneous scalar error (a loss). We implement a local, “three-factor” corticostriatal plasticity rule involving the presynaptic firing rate, a postsynaptic factor, and the unique dopamine concentration perceived by each striatal neuron. With this learning rule, we show that such a vector-valued feedback signal results in an increased capacity to learn a multidimensional series of real-valued outputs. Crucially, we demonstrate that this plasticity rule does not require precise nigrostriatal synapses but remains compatible with experimental observations of random placement of varicosities and diffuse volume transmission of dopamine.

## Significance

The striatum is thought to learn to select actions based on environmental feedback and rewards using a dopamine feedback signal. When the action is continuous and mutlidimensional, e.g., a reaching movement, it is beneficial that the dopamine feedback has the same dimensionality as the task. However, although the dopaminergic cell bodies exhibit multidimensional responses, it has been unclear whether they can be effectively used by the striatum given their broad and unspecific axonal arbors. We present a simplified simulation model where multidimensional dopamine activity improves learning compared to a single signal, despite the nonspecificity of the projections. Thus, we demonstrate the feasibility of multidimensional feedback using dopamine in the basal ganglia and make testable predictions.

The basal ganglia are thought to be the main locus of reinforcement learning (RL) in the brain (1). In particular, dopamine-modulated long-term plasticity in the corticostriatal synapses is crucial for learning and fine-tuning skilled movements based on environmental feedback (2). Combined with the striking observation that midbrain dopaminergic cells transmit a reward prediction error (RPE) to the striatum (3, 4), this has inspired a plethora of computational models of the basal ganglia implementing various forms of RL. Notably, however, virtually all these models assume the set of actions that can be selected—the action space in RL terminology—is small and discrete (see e.g., refs. 5–14). Practically, this means that each action can be exclusively represented by a disjoint group of striatal neurons, sometimes called action channels (15). At their core, in each of these models, there is some sort of competition between the action channels so that the selected action (or likely selected in probabilistic models) corresponds to the channel with the highest activity. This is consistent with a global RPE transmitted by dopamine that reinforces or depresses the corticostriatal synapses of the active channel.

However, there is now accumulating evidence that the action space of the basal ganglia is not small and discrete, but rather multidimensional and continuous (16–20). For a multidimensional output, a global RPE is not as effective at driving learning as it is for discrete action channels. For example, a mouse learning to reach for a food pellet may need to learn to control its paw in the *x*, *y*, and *z* directions. Intuitively, a single error, perhaps proportional to the final distance to the target, would be less efficient than having a three-dimensional error signal representing the error in the three directions.

More formally, producing a continuous and multidimensional output requires the basal ganglia to learn a function approximation rather than tabular values (21). Although simple function approximators (e.g., single-layer networks) can be successfully trained with a scalar global error, that strategy rapidly becomes untenable with increasing network depth and complexity (22). In practice, contemporary RL algorithms for continuous action spaces, e.g., A3C (23), DDPG (24) and PPO (25) rely on artificial neural networks trained with backpropagation to approximate the continuous policy.

Therefore, we asked whether it would be possible for dopamine to support function approximation learning in the basal ganglia by carrying a vector-valued feedback signal from the midbrain back to the striatum. Such a feedback signal would manifest in the VTA and SNc in terms of cell tunings to various task-related variables, consistent with recent observations (26–28). However, one apparent problem with dopamine transmitting a vector-valued error is that dopaminergic axons do not precisely target specific neurons in the striatum, but instead release dopamine from a large number of varicosities that can then diffuse over a short distance through extracellular space (29), thereby mixing any individual error components. In principle, one could imagine this problem being solved by representing each action dimension in a spatially compact region that receives its private dopamine channel (see refs. 30 and 31 for similar ideas). However, experimental evidence suggests that individual dopaminergic neurons (32) have mixed tuning rather than responding to a single task variable. Moreover, although there is a coarse-grain somatotopic organization of the striatum (33) as well as the substantia nigra (34), the axonal arborizations of individual SNc neurons are huge and cover large portions of the striatum (up to 5%; 35). Therefore, a large set of isolated parallel channels without cross-talk appears unlikely.

In this work, rather than separating the entire basal ganglia into fine-grained parallel channels, we propose that the mixing of multiple error components can be undone downstream from the striatum. In particular, we propose that if the striatofugal projections were to be subjected to similar systematic long-term plasticity as the corticostriatal projections, then we can make use of feedback alignment (36) to have striatum learn continuous outputs efficiently. We show that by using a stylized model of diffuse mixing of dopaminergic feedback as the random feedback, the Random Feedback Local Online (RFLO) learning rule (37) can be employed in a recurrent neural network model of the basal ganglia. This results in significantly improved learning compared to a model with homogeneous/scalar dopamine in the entire striatum. Thus, we connect two seemingly unrelated observations: heterogeneous dopamine response and the involvement of the striatum in learning complex and mutlidimensional continuous actions.

## Network Model

As a model of the basal ganglia learning a skilled movement (such as an animal reaching for a food pellet or pressing a lever), we constructed a task wherein a recurrent neural network must learn to repeatedly output a given trajectory in *d*-dimensional space. The output trajectory is defined as the activity of *d* readout neurons. In our idealized model of a small piece of basal ganglia, we take the readout population to be either the internal globus pallidus (GPi) or the substantia nigra pars reticulata (SNr) (see ref. 17, for experimental support).

**Fig. 1.**
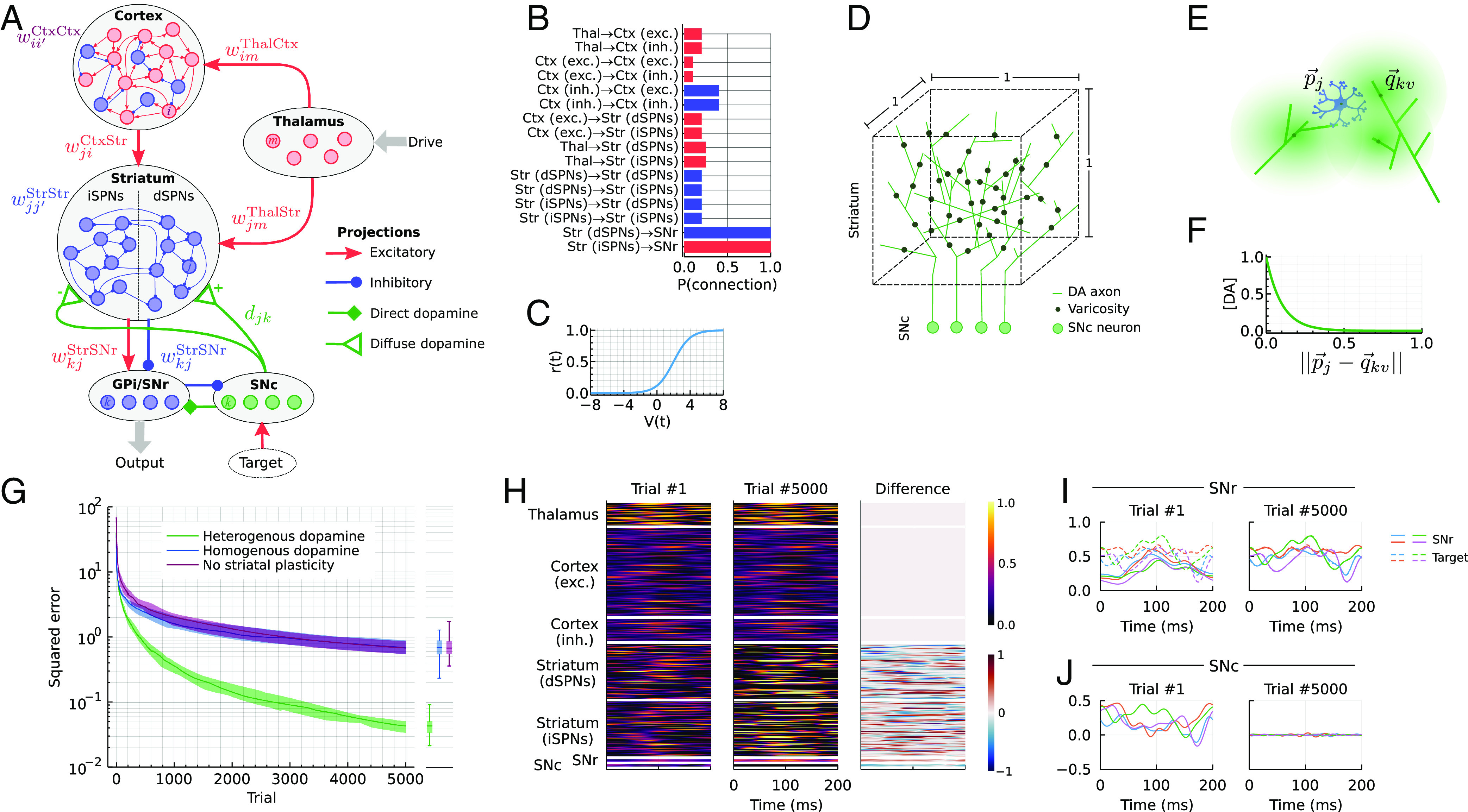
Heterogeneous dopamine improves learning in a RNN. (*A*) Network architecture. (*B*) Connection probability of a synapse for any pair of neurons in the network model. (*C*) Transfer function of each unit. (*D*) Illustration of placement of varicosities in a unit cube. (*E*) Illustration of striatal projection neurons (SPN) (located at $\vec{p_{j}}=\left[p_{x}^{j},p_{y}^{j},p_{z}^{j}\right]$) receiving a mix of dopamine from three close-by varicosities. (*F*) Relative dopamine concentration as a function of distance from the varicosity. (*G*) Convergence of the loss (L2) over trials for the model (heterogeneous dopamine) as well as two control models (homogeneous dopamine and no striatal plasticity). Each trial is one presentation of the pattern. Solid lines indicate the median of 25 runs; shaded areas show the first and third quartiles. Boxplots show median, quartiles, and min/max of the same 25 runs at the final (5,000th) training trial. (*H*) Example activity in the network in the first and last trials of training. (*I*) Comparison of the 4-dimensional readout (i.e., SNr/GPi activity) with the target in the first and last trial of training. (*J*) The difference between the readout and the target (i.e., SNc activity) in the first and last trial of training.

This striatum projects to the readout population (GPi/SNr) and receives excitatory inputs from two input populations: cortex and thalamus (Fig. 1*A*). The task of the network is to adjust the input and recurrent synaptic weights in the striatum so that the readout matches the desired *d*-dimensional target *T*(*t*) as closely as possible (Fig. 1*I*).

In the cortex and striatum, we model the subthreshold membrane potential *V*(*t*) of each neuron (or small group of neurons) as

[1] $$\begin{array}{c} \frac{dV(t)}{dt}=-\frac{1}{\tau_{m}}V\left(t\right)+\sum_{\mathrm{Pre}}\sum_{\mathrm{pre}}w_{post,pre}^{\mathrm{PrePost}}r_{\mathrm{pre}}^{\mathrm{Pre}}\left(t\right), \end{array}$$

where *τ*_*m*_ = 10ms is the membrane time constant. We write the synaptic weights as $w_{post,pre}^{\text{PrePost}}$ where Pre ∈ {Thal, Ctx, Str} is the presynaptic population and *pre* ∈ {*m*, *i*, *j*} is the index of the neuron in that population^*^. The firing rate *r*(*t*) of each neuron is calculated as:

[2] $$\begin{array}{c} r\left(t\right)=\phi \left(V(t)\right)=\frac{1}{1+e^{-V(t)+b}}, \end{array}$$

where the term *b* = 2 shifts the sigmoid to the right so that the firing rates are sparser when the inputs are balanced and the membrane potentials fluctuate around 0 (Fig. 1*C*). In the readout population (GPi/SNr), we index the neurons by *k* and let their firing rates be given by

[3] $$\begin{array}{c} r_{k}^{\mathrm{SNr}}\left(t\right)=\phi \left(\sum_{j=1}^{N^{\mathrm{Str}}}w_{\mathrm{kj}}^{\mathrm{StrSNr}}r_{j}^{\mathrm{Str}}\left(t\right)\right). \end{array}$$

The purpose of our model is to demonstrate that mixing of dopamine is not detrimental to vector-valued feedback and not to capture every detail of the basal ganglia. Nevertheless, to make sure the learning setup is fair, we added a number of biological constraints to the model. First, all connections except the readout are sparse, i.e., only a fraction of pairs of neurons are allowed to connect (Fig. 1*B*). Second, we required the signs of the weights to match the sign of the projection (excitatory or inhibitory) throughout learning (Fig. 1 *A* and *B*). For simplicity, we omitted the external globus pallidus (GPe) and subthalamic nucleus (STN) and modeled the indirect pathway as a direct excitatory projection from the striatum to GPi/SNr. The sign of dopamine-driven plasticity was reversed for the striatal projection neurons in the indirect pathway (iSPNs; Fig. 1*A*). Because of our focus on dopamine-dependent learning in the striatum, we only include the cortex as a reservoir of rich but task-aligned dynamics and do not consider any learning that might take place in the cortex itself. However, we include dopamine-dependent plasticity in all basal ganglia synapses: corticostriatal, thalamostriatal, striatostriatal, and striatofugal.

### Derivation of the Synaptic Plasticity Rule.

To construct normatively appropriate learning rules for the plastic synapses, we note the task is to minimize the loss

[4] $$\begin{array}{c} \ell \left(t\right)=\frac{1}{2}\sum_{k=1}^{d}{\left(r_{k}^{\mathrm{SNr}}\left(t\right)-T_{k}\left(t\right)\right)}^{2}. \end{array}$$

For the error to decrease over time, we would like the plasticity rule to change the weight of each corticostriatal synapse ($w_{\text{ji}}^{\text{CtxStr}}$) such that

[5] $$\begin{array}{c} \frac{dw_{\mathrm{ji}}^{\mathrm{CtxStr}}\left(t\right)}{dt}\propto -\frac{\partial \ell (t)}{\partial w_{\mathrm{ji}}^{\mathrm{CtxStr}}\left(t\right)}. \end{array}$$

In *SI Appendix*, Text S1, we show that by expanding this partial derivative with two simplifications, considering ℓ only at *t* (i.e., not backward or forward in time) and treating the firing rates of other striatal projection neurons (SPNs) as fixed as in ref. 37, we arrive at the following plasticity rule:

[6] $$\frac{\text{d}w_{ji}^{\text{CtxStr}}(t)}{\text{d}t}=-\alpha \gamma_{j}(t)p_{ji}(t),$$

[7] $$\tau^{\text{Str}}\frac{\text{d}p_{ji}}{\text{d}t}=-p_{ji}(t)+r_{j}^{\text{Str}}(t)\left(1-r_{j}^{\text{Str}}(t)\right)r_{i}^{\text{Ctx}}(t),$$

[8] $$\gamma_{j}(t)=\overset{d}{\underset{k=1}{\sum }}\epsilon_{k}(t)w_{kj}^{\text{StrSNr}}(t),$$

[9] $$\epsilon_{k}(t)=\left(r_{k}^{\text{SNr}}(t)-T_{k}(t)\right)r_{k}^{\text{SNr}}(t)\left(1-r_{k}^{\text{SNr}}(t)\right).$$

The plasticity rules for the thalamostriatal and striatostriatal synaptic weights are fully analogous. We interpret this plasticity rule in biological terms as follows. From Eq. **6**, we see that the weight update depends on a neuron-specific factor *γ*_*j*_ and a synapse-specific factor *p*_*ji*_. The latter is a low-pass filtered trace of a Hebbian-like product between pre- and postsynaptic firing. This could be identified as an eligibility trace (9, 38), and we note it could be represented, for example, by the local concentration of calcium in the spine.

The eligibility trace *p*_*ji*_ is multiplied by a “third factor” *γ*_*j*_. Experimental results suggest plasticity in corticostriatal synapses depends on three factors: presynaptic activity, postsynaptic activity, and dopamine (39, 40). Given that the two former are captured by *p*_*ji*_, we would like to associate *γ*_*j*_ in Eq. **6** with dopamine. If we assume the number of dopaminergic neurons to be the same as the number of read-out neurons,

[10] $$\begin{array}{c} N^{\mathrm{SNc}}=N^{\mathrm{SNr}}=d, \end{array}$$

we can assign *ϵ*_*k*_ to the firing rate $r_{\text{k}}^{\text{SNc}}$:

[11] $$\begin{array}{c} r_{k}^{\mathrm{SNc}}\left(t\right)=\epsilon_{k}\left(t\right). \end{array}$$

That is, we assume that the SNc has access to the vector-valued error (*Discussion*). Note that we can handle negative values of *ϵ*_*k*_ by loosely interpreting $r_{\text{k}}^{\text{SNc}}$ as the deviation from some baseline firing rate. This leaves just one problem: The coefficients used to sum the contribution of the dopaminergic cells in Eq. **8** should be the downstream striatofugal weights $w_{\text{kj}}^{\text{StrSNr}}$, which are not available to the corticostriatal synapses. However, following a similar derivation as above, Murray (37) showed that, if the readout weights $w_{\text{kj}}^{\text{StrSNr}}$ themselves are plastic, we can replace $w_{\text{kj}}^{\text{StrSNr}}$ in Eq. **8** with a random value at only a minor cost to the convergence of the loss, thanks to a phenomenon called feedback alignment (36). Therefore, we next asked whether the dopaminergic nigrostriatal projection could form such a random feedback matrix.

### Dopamine Diffusion.

To investigate whether dopaminergic feedback could be used to communicate the third factor needed for the corticostriatal plasticity, we set up a stylized model of dopamine diffusion. We assumed each striatal neuron had a position $\vec{p_{j}}=\left[p_{x}^{j},p_{y}^{j},p_{z}^{j}\right]$ where *p*_*x*_^*j*^, *p*_*y*_^*j*^, *p*_*z*_^*j*^ ∈ [0, 1]. Second, we assumed that each SNc neuron sent axonal projections that covered the entire cube and that axonal arbor of each SNc neuron has *N*^var^ = 10 varicosities randomly placed in the unit cube (Fig. 1*D*). Third, we assumed the dopamine released from each varicosity is proportional to the firing rate at the soma in the SNc and that the dopamine concentration decreases exponentially with distance from the varicosity. This gives the dopamine concentration *C*_*j*_(*t*) at striatal neuron *j* as

[12] $$\begin{array}{c} C_{j}\left(t\right)=\sum_{k=1}^{N^{\mathrm{SNc}}}\sum_{v=1}^{N^{\mathrm{Var}}}r_{k}^{\mathrm{SNc}}\left(t\right)\frac{1}{\lambda }e^{-\frac{\left|\right|\vec{p_{j}}-\vec{q_{\mathrm{kv}}}\left|\right|}{\lambda }}, \end{array}$$

where $\vec{q_{\mathrm{kv}}}=\left[q_{x}^{\mathrm{kv}},q_{y}^{\mathrm{kv}},q_{z}^{\mathrm{kv}}\right]$ is the position of the *v*th varicosity of SNc neuron *k*. *λ* controls the rate of decay with distance and was set to 0.1 (so that dopamine concentration decreases to 1/*e* ≈ 37% after diffusing a distance equivalent to 10% of the side of the cube). This model defines an effective nigrostriatal weight

[13] $$\begin{array}{c} d_{\mathrm{jk}}=\sum_{v=1}^{N^{\mathrm{Var}}}\frac{1}{\lambda }e^{-\frac{\left|\right|\vec{p_{i}}-\vec{q_{\mathrm{kv}}}\left|\right|}{\lambda }}, \end{array}$$

that does not vary with time, so we can write

[14] $$\begin{array}{c} C_{j}\left(t\right)=\sum_{k=1}^{N^{\mathrm{SNc}}}r_{k}^{\mathrm{SNc}}\left(t\right)d_{\mathrm{jk}}. \end{array}$$

Remember we assumed the vector-valued error *ϵ*_*k*_(*t*) is present in the SNc (Eq. **11**), and note the similarity between Eqs. **8** and **14**. If we introduce plasticity in the striatofugal projection, feedback alignment will cause $w_{\text{kj}}^{\text{StrSNr}}$(*t*)→*d*_*jk*_. Therefore, we again start with

[15] $$\begin{array}{c} \frac{dw_{\mathrm{kj}}^{\mathrm{StrSNr}}\left(t\right)}{dt}\propto -\frac{\partial \ell (t)}{\partial w_{\mathrm{kj}}^{\mathrm{StrSNr}}\left(t\right)}, \end{array}$$

and arrive at

[16] $$\begin{array}{c} \frac{dw_{\mathrm{kj}}^{\mathrm{StrSNr}}\left(t\right)}{dt}=-\beta \epsilon_{k}\left(t\right)r_{j}^{\mathrm{Str}}\left(t\right), \end{array}$$

with *ϵ*_*k*_(*t*)=*r*_*k*_^SNc^(*t*) as before and *β* = 10^−3^.

In summary, we set the corticostriatal plasticity update to

[17] $$\begin{array}{c} \frac{dw_{\mathrm{ji}}^{\mathrm{CtxStr}}\left(t\right)}{dt}=-\alpha p_{\mathrm{ji}}\left(t\right)\sum_{k=1}^{N^{\mathrm{SNc}}}r_{k}^{\mathrm{SNc}}\left(t\right)d_{\mathrm{jk}}, \end{array}$$

where *d*_*jk*_ is given by Eq. **13** and *p*_*ji*_(*t*) is given by Eq. **7**. We set *α* = −2.5 ⋅ 10^−2^ for direct pathway neurons and *α* = 2.5 ⋅ 10^−2^ for indirect pathway neurons to capture the different effects of D1 and D2 receptors. The minus sign helps $w_{\text{kj}}^{\text{StrSNr}}$(*t*)→*d*_*jk*_ for direct pathway striatal neurons, because for these neurons $w_{\text{kj}}^{\text{StrSNr}}$(*t*)< 0. We used analogous plasticity rules for thalamostriatal and striatostriatal connections.

## Learning with Vector-Valued Dopamine Feedback

Having set up the model, we simulated the dynamics for 5,000 presentations of the target output (Fig. 1*H*). Each target was a 4-dimensional 200-ms time series drawn from a Gaussian process (Fig. 1I; see *Methods*). In the first trial, the output does not match the target (Fig. 1 *I*, *Left*), but after 5,000 trials, the plasticity rules have driven the network to produce GPi/SNr output that closely matches with the targets (Fig. 1*I*, *Right*). This is achieved both by plasticity in the readout population (GPi/SNr; Eq. **17**) and plasticity in the striatum that adapts the SPN firing rates (Eq. **17**; Fig. 1*H*).

We next asked how this learning depends on the nature of the dopamine feedback. It is well known that for recurrent networks with rich dynamics, plasticity in the readout is sufficient to learn complex patterns (41, 42). Therefore, we first compared our model to a reduced model that only had plasticity in the readout (striatofugal) projection according to Eq. **16**, i.e., no plasticity in the striatum. We found that learning with dopamine feedback was faster (Fig. 1*G*). Next, we compared our model to a model in which each SPN at every time point received the same dopamine feedback, i.e., the striatum receives a homogeneous, scalar dopamine signal. Strikingly, this model performed no better than the reduced model that only has learning in the readout layer (Fig. 1*G*). The increase in learning performance persisted with different sizes of the striatal (*SI Appendix*, Fig. S1*A*) and the readout (*SI Appendix*, Fig. S1*B*) populations, as well as for faster and slower timescales of the target (*τ*_task_ in Eq. **22**; *SI Appendix*, Fig. S1*C*). These observations show that vector-valued dopamine feedback is crucial for the improvement in learning.

### The Improvement in Learning Is because of Feedback Alignment.

**Fig. 2.**
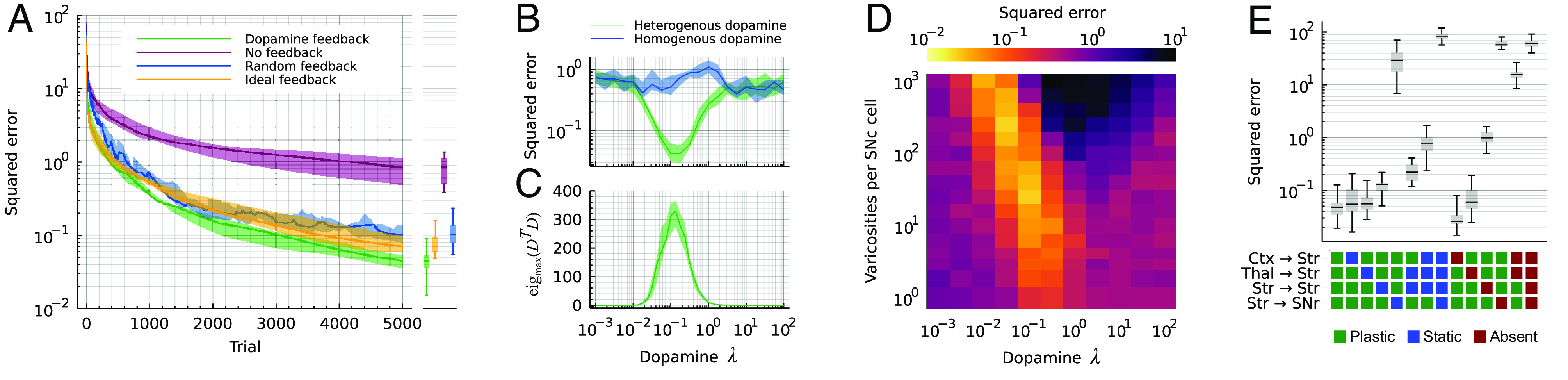
Learning improvement depends on feedback alignment. (*A*) Convergence of the loss (L2) for different types of feedback: dopamine feedback as described by our model, no feedback at all (effectively making all synapses except the striatofugal static), random feedback where the elements of the feedback matrix *D* are shuffled randomly and ideal feedback where *D* = *W*_StrSNr_^*T*^. (*B*) Squared error after 5,000 trials as a function of the spatial constant of dopamine diffusion (*λ*). *λ* was measured relative to the side of the cube in Fig. 1*D*. (*C*) Linear independence of the rows of *D* as a function of the dopamine spatial constant *λ*. (*D*) Squared loss after 5,000 trials as a joint function of the dopamine spatial constant *λ* and the number of varicosities per SNc cell. (*E*) Squared error after 5,000 trials when some of the four plastic projections are impaired: either set to static, i.e., no plasticity, or removed from the model altogether (absent). See *SI Appendix*, Fig. S1*D* for the complete set of manipulations. (All panels) errors are reported as the median over 25 trials. Shaded areas indicate the first and third quartiles of the 25 trials. Boxplots show median, first, and third quartiles; whiskers indicate min and max.

Next, we further explored the conditions during which vector-valued feedback improved learning of the targets. First, we considered two additional alternative models: i) the feedback is random, i.e., we still have vector-valued feedback, but shuffle the coefficients *d*_*jk*_ of Eq. **14** and ii) we use the “ideal” feedback *d*_*jk*_ = $w_{\text{kj}}^{\text{StrSNr}}$(*t*). Note that locking the feedback weights to the feed-forward in the second model means the feedback matrix is time-dependent. Performance of both of these models was similar to our dopamine model (Fig. 2*A*). This suggests that the main criterion for the feedback to be effective is that feedback matrix *D* = [*d*_*jk*_] is nondegenerate.

With this hypothesis in mind, we tested varying the spatial scale *λ* of dopamine diffusion (Eq. **13**). Note that this spatial scale was measured as a fraction of the side of the cube (Fig. 1*D*). When *λ* ≪ 1, almost no dopamine reaches any SPN from the varicosities, and the network reverts to the No feedback control model (Fig. 2*B*). On the other hand, when *λ* ≫ 1, the dopamine from each varicosity covers the entire cube so that all SPNs effectively receive the sum of the dopamine released anywhere. This causes the network to revert to the Homogeneous dopamine control model. In between these two extremes, where the dopamine scale is intermediate, there is a sweet spot where each SPN receives dopamine corresponding to a unique random linear projection of the 4-dimensional error (Fig. 2*C*). In this regime, the benefit of the feedback is the largest (Fig. 2*B*). Finally, we also varied the number of varicosities *N*^Var^ in Eq. **13** and found that with a larger number *N*^Var^, a smaller spatial scale *λ* becomes viable (Fig. 2*D*). This is also consistent with the creation of a nondegenerate *D*.

To illustrate the importance of striatofugal plasticity for learning, we simulated the network model with “lesioned” basal ganglia projections by either removing the plasticity (static) or clamping them to 0 (absent). As expected, when the plasticity of the striatofugal projection was turned off, no feedback alignment could take place and the striatal plasticity could not contribute to learning the targets (Fig. 2*E*, Str→SNr). Turning off plasticity of the striatal projections (corticostriatal, thalamostriatal, and striatostriatal) on the other hand has a more moderate impact. This is because even with all of them fixed, we can still have echo-state-like learning in the striatofugal weights (see the No feedback null model in Figs. 1*G* and 2*A*). Similarly as when fixing the weights, removing either the cortical or the thalamic projection does not change the eventual error much, as both projections play similar and mostly interchangeable roles in our model, whereas removing both silences the striatum completely and hence gives a very large error (Fig. 2*E*). See *SI Appendix*, Fig. S1*D* for a systematic investigation of how removing or blocking plasticity on different projections affects learning.

### Fast Synaptic Dynamics Can Compensate for Slow Dopamine.

So far, we have assumed that dopamine is diffuse in space but delivered instantly to the receiving SPNs. While this allows the synaptic weights to be updated correctly on every time step, it neglects the temporal dynamics of dopamine release, diffusion, reuptake, etc. A faithful quantitative model of these processes is beyond the scope of our abstract rate network, but it is nevertheless important to determine how dependent our dopamine-based learning rule is on the assumption of instantaneous dopamine release. We simplified all temporal dynamics of dopamine into a simple exponential low-pass filter with time constant *τ*_DA_. That is, we changed the equation for dopamine concentration at striatal neuron *j* (Eq. **12**) to

[18] $$\begin{array}{c} C_{j}^{\prime }\left(t\right)=\int_{0}^{t}e^{\frac{t^{\prime }-t}{\tau_{\mathrm{DA}}}}C_{j}\left(t^{\prime }\right)dt^{\prime }. \end{array}$$

**Fig. 3.**
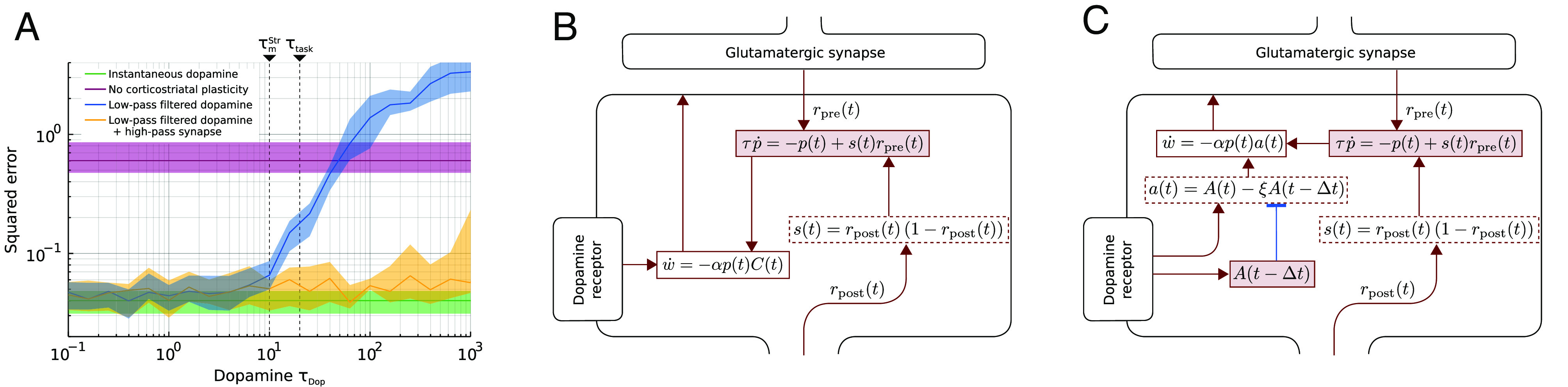
Slow dopamine dynamics. (*A*) When the dopamine signal is low-pass filtered with a time constant slower than the time constant of the target output, the error (blue) increases. For comparison, the errors of previous models with instantaneous (green) and absent (purple) dopamine are also shown (these do not depend on the *τ*_Dop_). Finally, the orange line shows the error when using the extended synaptic dynamics shown in panel *C*. All errors are reported as the median over 25 trials. Shaded areas indicate the first and third quartiles of the 25 trials. (*B*) The synaptic dynamics of the standard RFLO learning rule. (*C*) Adding an extra forward-inhibition motif to the synaptic dynamics to compensate for temporal smoothing of dopamine.

The blue line in Fig. 3*A* shows the resulting error after 5,000 trials for a range of values of *τ*_DA_. When *τ*_DA_ is much faster than the time constant of the task (here *τ*_task_ = 20ms), the error (Fig. 3*A*, blue line) is similar to the earlier, instantaneous dopamine model (Fig. 3*A*, green line). However, for slower *τ*_DA_, the error increases and even surpasses the null model with no striatal plasticity (Fig. 3*A*, purple line).

We next asked what would be needed to rescue the learning performance in face of slow dopamine dynamics. One possible solution would be that each synapse high-pass filters its local dopamine concentration. Such high-pass filtering can be done by assuming a feed-forward inhibition motif as the first step in the biochemical pathway triggered by dopamine (Fig. 3*C*). In the ideal case, one biochemical node in each synapse is tracking dopamine concentration one time step ago, and another calculates the difference

[19] $$\begin{array}{c} a(t)=A(t)-\xi A(t-\Delta t). \end{array}$$

Furthermore, if we choose *A*(*t*)=*C*_*j*_′(*t*)/(1 − *ξ*) and $\xi =e^{-\frac{\Delta t}{\tau_{\mathrm{DA}}}}$ we get *a*(*t*) ≈ *C*_*j*_(*t*) and we can use the same RFLO learning rule as before but with *a*(*t*) instead of *C*_*j*_(*t*) (compare Figs. 3 *B* and *C*).

We verified this idea by introducing the synapse model in Fig. 3*C* in all striatal synapses and then again plotting the error after 5,000 trials. As predicted by the ideal choice of *ξ*, the error was consistently similar to instantaneous even for large *τ*_DA_ (Fig. 3*A*, yellow line).

### Spatial Dopamine Induces Spatial Structure of SPN Responses.

**Fig. 4.**
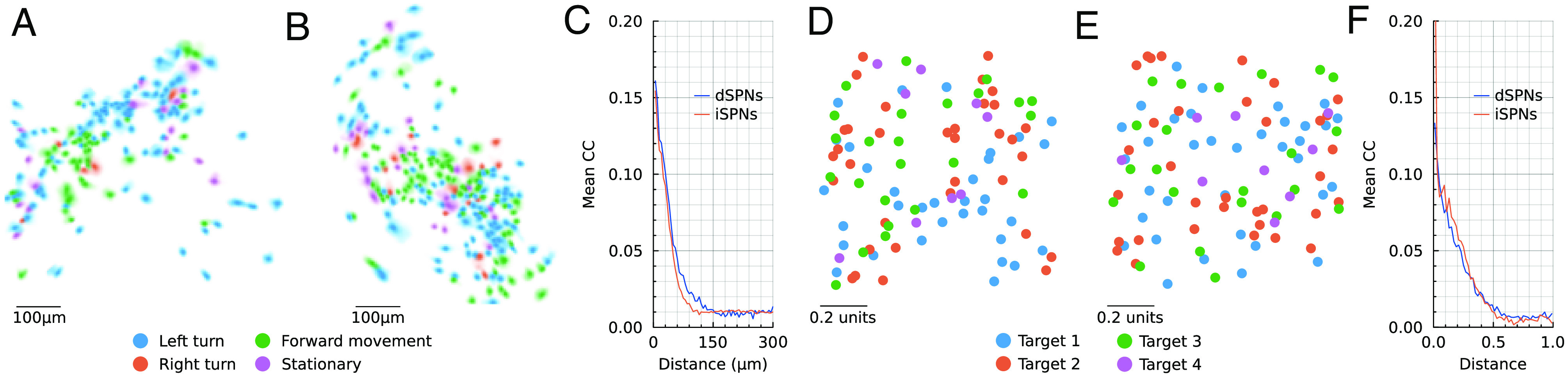
Spatial correlations. (*A*) Calcium-imaging (1-photon) footprints of dSPNs in the *Right* dorsomedial striatum of a mouse exploring an open field. The footprints of the neurons are colored according to which basic movement they respond most strongly to. The most common movement is turns contralateral (*Left*) to the recorded hemisphere (*Right*), but all four movements are represented by some neurons. (*B*) Same as *A*, but iSPNs from a different mouse. (*C*) Mean Pearson’s correlation coefficient between the deconvolved calcium traces of pairs of SPNs as a function of the distance between the center of their footprints in the field-of-view. Means were calculated by pooling data from 7 (dSPNs) and 8 (iSPNs) recording sessions. Data for *A*–*C* was adapted from ref. 47. (*D*) The *x* and *y* coordinate of simulated dSPNs, with colors indicating which target dimension it was most correlated with after 5,000 trials of training. The *z* coordinate was omitted, as if the cells are orthographically seen from above. (*E*) The iSPNs from the same simulation as in (*D*). (*F*) Mean Pearson’s correlation coefficient between the firing rates of pairs of SPNs in the simulation as a function of the distance between them. The means are calculated across all pairs of SPNs pooled from 1,000 simulations. Distance is measured relative to the side of the cube.

A ubiquitous observation in 1-photon calcium imaging in rodents is that SPNs that are active during some action tend to be spatially close to other SPNs responding to the same action (43–47, Fig. 4 *A* and *B*). At the same time, the actions are not represented by entirely isolated clusters because examples of SPNs preferring any given action can always be found in all parts of the miniscopes’ fields-of-view (44–47, Fig. 4 *A* and *B*). Mechanistically, this arrangement could be simply explained by spatially neighboring SPNs sometimes receiving inputs from shared cortical or thalamic axons, as well as from other SPNs or interneurons close-by. However, the algorithmic and computational significance of this spatial arrangement has been controversial, with some authors interpreting it in favor of spatially compact action channels (43, 46) and some authors in favor of a distributed representation of actions (44, 47).

Therefore, we asked whether the spatial structure of dopamine diffusion in our model was sufficient to induce the weak but significant distance-dependent correlation between pairs of SPNs together with a spatially distributed representation of the action. We found both phenomena (Fig. 4 *D*–*F*). In our model, target dimension are equivalent to the “actions”. Consistent with the experimental data, we found examples of both dSPNs and iSPNs preferring all four target dimensions in all parts of a constructed field-of-view (Fig. 4 *D*–*E*). In addition, correlation between SPN firing rates (*r*_*j*_^Str^(*t*)) was around 0.15 for very close SPNs and decayed to zero for SPNs further apart (Fig. 4*F*), which closely resembles the shape of distance-dependent correlation between deconvolved calcium fluorescence traces (Fig. 4*C*). Note that we only used the spatial position of the SPNs (${\vec{p}}_{j}$) to construct the matrix *D* (Eq. **13**) and not to initialize the synaptic weights. Therefore, Fig. 4*F* shows exclusively the spatial structure induced by dopamine-dependent plasticity and we note that this is sufficient to reach experimental-level strength of the correlations. This is not the case for a much smaller or larger dopamine spatial constant *λ* (*SI Appendix*, Fig. S2). Nevertheless, we note that space-dependent connectivity probabilities and shared inputs will also likely contribute to the spatial dependence of the correlations in real experiments. In conclusion, we have demonstrated that the experimentally observed level of spatial correlations is compatible also with a distributed representation of actions in the striatum and could be a consequence of dopamine being used as a vector-valued error.

## Discussion

The broad, unspecific dopaminergic axonal projections have been argued to only allow for the transmission of a scalar homogeneous feedback signal (48). Here, we provide a tenable counterexample of this view, even if speculative and highly idealized. We demonstrate that in a reduced model of a piece of basal ganglia that a heterogeneous, vector-valued feedback signal could in fact be transmitted by dopamine, even if the dopaminergic projections in the striatum are random. We have identified four key requirements for effective use of vector-valued dopamine, which also serve as predictions that can be verified experimentally:

1. At least one projection downstream of the striatum must be plastic.
2. The vector-valued error must be available to both the dopaminergic (here, SNc) and the readout (here, SNr) populations.
3. Striatal dopamine dynamics must be at least as fast as the targeted movements, or, alternatively, be high-pass filtered by feed-forward inhibition in the synaptic biochemical pathways.
4. The dopamine received by each SPN must be sufficiently independent, or, stated formally, the effective connectivity matrix arising from summing contribution of individual varicosities (Eq. **13**) must not be degenerate.

Direct experimental evidence for any downstream plasticity in the basal ganglia (Requirement 1) is scarce, but a recent study by González-Rodríguez et al. (49) showed that dopamine depletion in the SNr plays a larger role than striatal dopamine in producing motor deficits in Parkinson’s disease. This is qualitatively consistent with the relative importance of striatonigral over corticostriatal plasticity in our model (Fig. 2*F*). However, we note that although we placed this plasticity in the striatofugal projection(s), it could in principle also be met by plasticity in the nigrothalamic or nigrocollicular projections.

A vector-valued error would likely appear as tunings to various motor and task variables in experimental animals (Requirement 2), especially in the phase before the animals are so overtrained their error is zero. Indeed, cells in the SNr (17, 26, 50) as well as the SNc (27, 32, 51, 52) respond to a plethora of behavioral and task variables. We deliberately excluded the details of how this error may be computed in the brain, but we speculate at least three possible algorithmic ways in which it could appear:

1. Brain regions such as the motor cortex or cerebellum could have a forward model of the world as well as the target and thus can directly compute the error and send it to the midbrain.
2. The brain could be wired as a set of hierarchical control loops, in which each loop provides the target for the level below (as proposed by ref. 16). Each such loop could stretch throughout the cortex and basal ganglia.
3. If the executed action has more variability than the command read out by the SNr, the policy gradient theorem (21) states that the gradient for the update should be
  [20] $$\begin{array}{c} r^{\mathrm{SNc}}\left(t\right)\propto \delta \left(t\right)\nabla \ln p\left(a\left(t\right)|r^{\mathrm{SNr}}\left(t\right)\right), \end{array}$$
  where *a* is the vector-valued action taken, *δ* is the temporal difference (TD) error as predicted by a critic, and *p* is the probability density function of *a*. Note that this suggests that SNc cells should fire proportionally to both the TD error and to (the gradient of) some behavioral variables, which could explain why many SNc cells appear tuned to both (53, 54). Lindsey and Litwin-Kumar (55) have proposed that the dorsal striatum could make use of such a policy gradient but nonetheless argue that dopamine itself is a scalar proportional to the squared norm of the policy gradient.

Importantly, none of these ways requires the vector-valued error to be provided directly from a supervisor external to the brain. Including one or more brain regions that use some of these principles to translate external rewards and internal goals into a vector-valued error or policy gradient will be a critical future extension of our model.

There are several ways the brain could implement filters that allow extraction of faster fluctuations in dopamine concentration (Requirement 3). Our example in Fig. 3 is highly idealized and makes arguably unfair use of our idealized perfect exponential decay of the dopamine. In reality, the journey of dopamine molecule from a varicosity to a dopamine receptor depends on the local geometry and dopamine reuptake so that the dependence on both time and distance is most likely complicated and nonlinear (although these effects might be less pronounced at very short distances; 56, 57). Nevertheless, evolution has had a good opportunity to tweak the biochemical pathways to compensate for these effects as far as permitted by the signal-to-noise ratio. Whether this is tenable in a realistic model of dopamine diffusion and biochemical cascades remains an open question, but we predict that there is at least one node in the biochemical cascade of dopamine-induced synaptic plasticity that is sensitive to fast fluctuations in local dopamine concentration.

The spatial frequency of the dopamine landscape in the striatum must be high enough so that even neighboring SPNs do not sense the exact same dopamine concentration (Requirement 4). This can be achieved by having a short spatial constant of dopamine diffusion, and possibly compensating with a larger number of varicosities (Fig. 2*D*). Consistent with our model, Cragg and Rice (56) estimated the diffusion distance of dopamine following release to a few microns.

The main goal of our work was to demonstrate that the broad and unspecific nigrostriatal dopaminergic projection can in principle transfer a usable vector-valued error to the striatum; our ambition was not to provide a complete biological account of the process. For this reason, there are many likely very important features of basal ganglia anatomy and physiology we did not include, for example, dorsolateral/dorsomedial functional division in the striatum (58), the different roles of the matrix and the striosome (59), axonally initiated dopaminergic release by cholinergic interneurons (60, 61), saturating dopamine receptors (57), etc., Similarly, our primary goal was not to introduce a new algorithm for training recurrent neural networks; the network setup and plasticity rule is an application of the RFLO rule (37). Nevertheless, we show that the RFLO rule is applicable in a basal ganglia-like network with multiple inhibitory synapses and with our reduced model of dopamine feedback and propose vector-valued error feedback as a candidate functional role of dopamine.

Whether the striatum actually takes advantage of the vector-valued dopamine in the manner we proposed here could in principle be tested by building on existing experiments. For example, Bova et al. (62) have shown that optogenetically stimulating SNc of rats during a reaching task results in impairment also in subsequent, nonstimulated trials. This demonstrates the key role of midbrain dopamine in motor learning. A similar, but technically more challenging, experiment could be used to test the importance of the vector-valued nature of the dopamine signals in the striatum. If two opsins with opposing effects are simultaneously expressed in the dopaminergic axons in the striatum, they could be carefully stimulated using two-colored patterned illumination so that some terminals are excited and some are inhibited, while the net dopamine release in the area remains the same. Our prediction is that in this scenario, motor learning is impaired because even though the precise temporal nature of the net (that is, scalar) dopamine signal remains intact, its vector-valued nature is scrambled.

Previous proposals for use of heterogeneous dopamine (30, 31) assume that the heterogeneous responses of dopaminergic cells are transmitted to the striatum through private parallel channels without any cross-talk. However, this is not easily reconciled with functional and anatomical findings (*Introduction*). Similarly, Gardner et al. (63) have proposed that dopamine conveys a vector-valued sensory prediction error (SPE) that can be used to update a parameterized successor representation. Although such an SPE could explain the heterogeneous firing pattern of midbrain dopaminergic neurons, it would require the vector-valued signal to be transmitted intact to the receiving area (striatum). Another proposed use of heterogeneous firing in the midbrain dopaminergic neurons is to support a distributional coding of value (64). However, a distributional value code only explains different gains in the coding of the reward prediction error, not why the neurons respond to nonrewarded task variables. Nevertheless, it is entirely possible that the brain simultaneously employs a distributional value code (perhaps most strongly in the VTA) for a “critic” subregion and a vector-valued error code (perhaps most strongly in the SNc) for an “actor” subregion.

In conclusion, we propose that the experimental observation of heterogeneous responses of dopamine cells (26–28) can represent a vector-valued error. By providing this type of error, the SNc supports the basal ganglia learning to select actions from a continuous action space in continuous time, thereby providing the animal with vital behavioral flexibility, control, and adaptability.

## Materials and Methods

The dynamics of neurons, network structure, and learning rule is already described in the Results section. Here, we describe only the technical details needed to run the simulations.

### Network Simulations.

The network was simulated in a custom simulator written in Julia (65). The dynamics were simulated with forward-Euler with dt = 1 ms. The number of units in each population is shown in *SI Appendix*, Table S1.

Simulations consisted of multiple trials concatenated after each other without any reset of the network in between. The current time in the current trial was signaled to the network by setting the thalamic firing rates to

[21] $$\begin{array}{c} r_{m}^{\mathrm{Thal}}\left(t\right)=\phi \left(A_{m}\cos\frac{2\pi t}{T}+B_{m}\sin\frac{2\pi t}{T}\right), \end{array}$$

where *T* = 200 ms is the duration of a single trial, and *A*_*m*_ and *B*_*m*_ are constants drawn randomly from a circle with radius 4 (i.e., $A_{m}^{2}+B_{m}^{2}=4^{2}$ for all *m*). *ϕ* is the logistic transfer function (Eq. **2**).

### Initializing the Weights.

For each pair of cells in each projection, there was a fixed probability (Fig. 1*B*) of a synapse being inserted. If a synapse was inserted, its weight was drawn from a uniform distribution $\left[0,w_{\max}/\sqrt{N_{\mathrm{post}}}\right]$ (*SI Appendix*, Table S2) and then multiplied by −1 for the inhibitory projections. The weights in *SI Appendix*, Table S2 were chosen for the network to have close to chaotic trajectories before training.

After all synapses were created, the sum of the weight of all incoming synapses was calculated for each neuron. If this sum was greater than 0, all the inhibitory synapses were slightly increased so that the new sum was exactly 0. Conversely, if the sum was less than 0, all the excitatory weights were slightly increased to reach sum 0. This ensured that each neuron had roughly balanced excitation and inhibition, which in turn created rich dynamics from the start.

### Target Signals.

The targets were drawn from a Gaussian process with mean 0.5 and variance given by

[22] $$\begin{array}{c} \sigma^{2}\left(t_{1},t_{2}\right)=0.15^{2}\exp\left(-\frac{\delta {\left(t_{1},t_{2}\right)}^{2}}{\tau_{\mathrm{task}}^{2}}\right), \end{array}$$

where *δ*(*t*_1_, *t*_2_) is the smallest difference between *t*_1_ and *t*_2_ when including wrap-around, i.e.

[23] $$\begin{array}{c} \delta \left(t_{1},t_{2}\right)=\min(|t_{1}-t_{2}\left|,\right|t_{1}-t_{2}+T|,|t_{1}-t_{2}-T\left|\right), \end{array}$$

where *T* = 200 ms is the duration of a single trial. The periodic kernel is to avoid discontinuities when running consecutive trials without resetting the network. For all experiments in the main figures, *τ*_task_ = 20 ms.

## Supplementary Material

Appendix 01 (PDF)

## Data Availability

Code for reproducing the simulations can be found at GitHub at https://github.com/emiwar/diffuse-dopamine-rnn (66).

## Footnotes

^*^We use the order *post*, *pre* for the subscript to follow the matrix-friendly convention where the first letter indexes the row and the second the column.
