# Supplementary material for: Feasibility of dopamine as a vector-valued feedback signal in the basal ganglia
Source: Proc Natl Acad Sci U S A. 2023 Aug 1;120(32):e2221994120. doi: 10.1073/pnas.2221994120 (PMC10410740; doi:10.1073/pnas.2221994120)
Supplement: Supplementary file 1 — Appendix 01 (PDF) [file pnas.2221994120.sapp.pdf]

Supporting Information for

Feasibility of dopamine as a vector-valued feedback signal in the basal ganglia

Emil Wärnberg and Arvind Kumar

Corresponding author: Arvind Kumar
E-mail: arvkumar@kth.se

This PDF file includes:

Supporting text
Figs. S1 to S2
Tables S1 to S2
SI References

Supporting Information Text

1. Derivation of the plasticity rule

We begin by restating the network equations. First, we write out the three inputs (cortex, thalamus and recurrent striatum) as

$$I_j(t') = \sum_{i=1}^{N^{\mathrm{Ctx}}} w_{ji}^{\mathrm{CtxStr}}(t') r_i^{\mathrm{Ctx}}(t') + \sum_{m=1}^{N^{\mathrm{Thal}}} w_{jm}^{\mathrm{ThalStr}}(t') r_m^{\mathrm{Thal}}(t') \tag{1}$$

$$+ \sum_{j'=1}^{N^{\mathrm{Str}}} w_{jj'}^{\mathrm{StrStr}}(t') r_{j'}^{\mathrm{Str}}(t') \tag{2}$$

Then we rewrite the membrane equation as an integral

$$V_j^{\mathrm{Str}}(t) = \int_0^t \exp\left(\frac{t'-t}{\tau^{Str}}\right) I_j(t')\mathrm{d}t' \tag{3}$$

$$r_j^{\mathrm{Str}}(t) = \phi\left(V_j^{\mathrm{Str}}(t)\right) \tag{4}$$

Since we treat SNr as a read-out layer, we let $\tau^{\mathrm{SNr}} \to 0$ so that

$$V_k^{\mathrm{SNr}}(t) = B^{\mathrm{SNr}} + \sum_{j=1}^{N^{\mathrm{Str}}} w_{kj}^{\mathrm{StrSNr}}(t) r_j^{\mathrm{Str}}(t) \tag{5}$$

$$r_k^{\mathrm{SNr}}(t) = \phi\left(V_k^{\mathrm{SNr}}(t)\right) \tag{6}$$

The instantaneous loss is

$$\ell(t) = \frac{1}{2}\sum_{k=1}^{d}\left(r_k^{\mathrm{SNr}}(t) - T_k(t)\right)^2 \tag{7}$$

where $T(t) \in \mathbb{R}^d$ is the target output at time t. To greedily (i.e. not considering earlier or later losses) minimize $\ell(t)$ (see 1) we want to have

$$\frac{\mathrm{d}w_{ji}^{\mathrm{CtxStr}}(t)}{\mathrm{d}t} \propto -\frac{\partial \ell(t)}{\partial w_{ji}^{\mathrm{CtxStr}}(t)} \tag{8}$$

Expanding this taking a partial derivative where $r_{j'}(t)$ is fixed for $j' \neq j$:

$$-\frac{\partial \ell(t)}{\partial w_{ji}^{\mathrm{CtxStr}}(t)} = -\sum_{k=1}^{d}\left(r_k^{\mathrm{SNr}}(t) - T_k(t)\right)\frac{\partial r_k^{\mathrm{SNr}}(t)}{\partial w_{ji}^{\mathrm{CtxStr}}(t)} \tag{9}$$

$$= -\sum_{k=1}^{d}\underbrace{\left(r_k^{\mathrm{SNr}}(t) - T_k(t)\right)\phi'\left(V_k^{\mathrm{SNr}}(t)\right)}_{\epsilon_k(t)}\frac{\partial v_k^{\mathrm{SNr}}(t)}{\partial w_{ji}^{\mathrm{CtxStr}}(t)} \tag{10}$$

$$= \underbrace{-\sum_{k=1}^{d}\epsilon_k(t) w_{kj}^{\mathrm{StrSNr}}(t)}_{\gamma_j(t)}\frac{\partial r_j^{\mathrm{Str}}(t)}{\partial w_{ji}^{\mathrm{CtxStr}}(t)} \tag{11}$$

$$= -\gamma_j(t)\phi'\left(V_j^{\mathrm{Str}}(t)\right)\frac{\partial V_j^{\mathrm{Str}}(t)}{\partial w_{ji}^{\mathrm{CtxStr}}(t)} \tag{12}$$

$$= -\gamma_j(t)\int_0^t \exp\left(\frac{t'-t}{\tau^{\mathrm{Str}}}\right)\phi'\left(V_j^{\mathrm{Str}}(t')\right)\frac{\partial I_j(t')}{\partial w_{ji}^{\mathrm{CtxStr}}(t)}\mathrm{d}t' \tag{13}$$

$$= -\gamma_j(t)\underbrace{\int_0^t \exp\left(\frac{t'-t}{\tau^{\mathrm{Str}}}\right)\phi'\left(V_j^{\mathrm{Str}}(t')\right) r_i^{\mathrm{Ctx}}(t')\mathrm{d}t'}_{p_{ji}(t)} \tag{14}$$

$$= -\gamma_j(t) p_{ji}(t) \tag{15}$$

Furthermore, note that

$$\phi'(V) = -\frac{-e^{-V+b}}{(1+e^{-V+b})^2} = \frac{1}{1+e^{-V+b}}\frac{(1+e^{-V+b})-1}{1+e^{-V+b}} \tag{16}$$

$$= \frac{1}{1+e^{-V+b}}\left(1 - \frac{1}{1+e^{-V+b}}\right) = \phi(V)\left(1-\phi(V)\right) \tag{17}$$

In summary, we get the following update rule for the corticostriatal synaptic weights

$$\epsilon_k(t) = \left(r_k^{\mathrm{SNr}}(t) - T_k(t)\right) r_k^{\mathrm{SNr}}(t) \left(1 - r_k^{\mathrm{SNr}}(t)\right) \quad [18]$$

$$\gamma_j(t) = \sum_{k=1}^{d} \epsilon_k(t) w_{kj}^{\mathrm{StrSNr}}(t) \quad [19]$$

$$\tau^{\mathrm{Str}} \frac{\mathrm{d}p_{ji}}{\mathrm{d}t} = -p_{ji}(t) + r_j^{\mathrm{Str}}(t) \left(1 - r_j^{\mathrm{Str}}(t)\right) r_i^{\mathrm{Ctx}}(t) \quad [20]$$

$$\frac{\mathrm{d}w_{ji}^{\mathrm{CtxStr}}(t)}{\mathrm{d}t} = -\alpha \gamma_j(t) p_{ji}(t) \quad [21]$$

The update rules for thalamostriatal (w^{ThalStr}) and striatostriatal (w^{StrStr}) have the same form and are derived in the same way. The striatofugal weight plasticity is

$$\frac{\mathrm{d}w_{kj}^{\mathrm{StrSNr}}(t)}{\mathrm{d}t} = -\beta \epsilon_k(t) r_j^{\mathrm{Str}}(t) \quad [22]$$

with the same ϵ_k as above.

References

1. JM Murray, Local online learning in recurrent networks with random feedback. *eLife* **8**, 1–25 (2019).

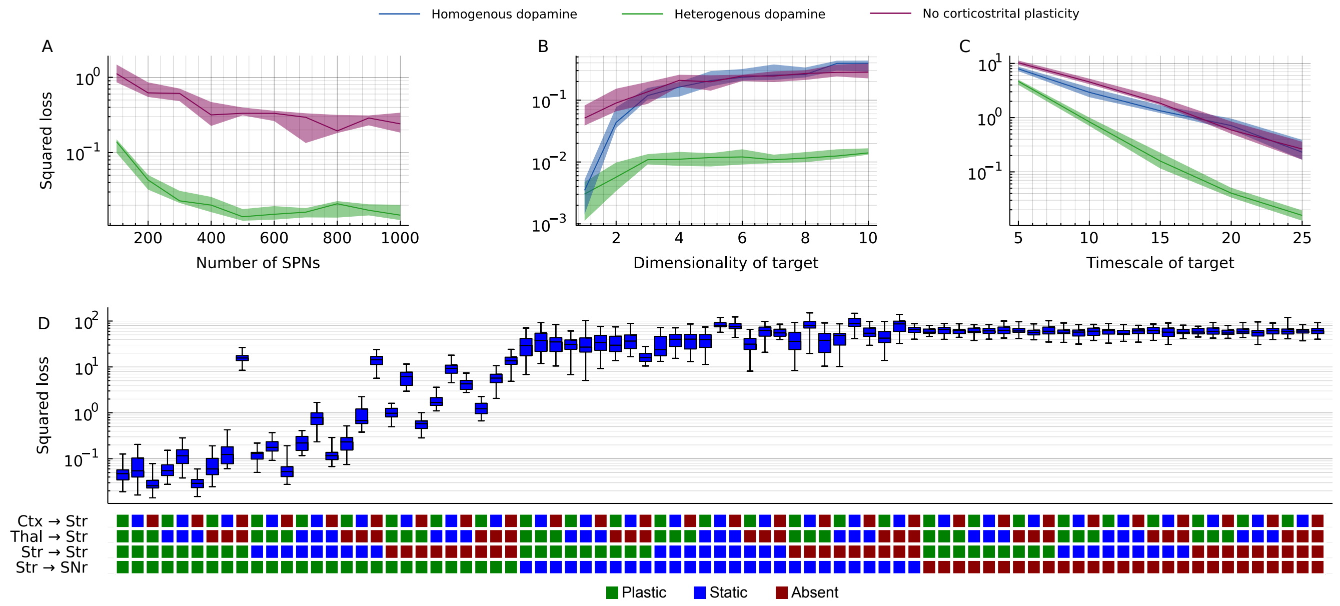

Fig. S1. Influence of each projection to the loss. (A) Mean squared error after 5000 training trials for different sizes of the striatal population. (B) Mean squared error for increasing values of $N^{\mathrm{SNr}} = N^{\mathrm{SNc}} = d$. (C) Mean squared error for increasing values of τ_{task}. (D) The network model was run with impairments to some of the connections. Either the plasticity was removed so that the synaptic weights of the connection were fixed to their starting weights ("static"), or the synapses were removed altogether ("absent"). Squared error is measured as the error after 5000 trials. Boxplots show median and quartiles across 25 runs; whiskers indicate min and max of the 25 runs.

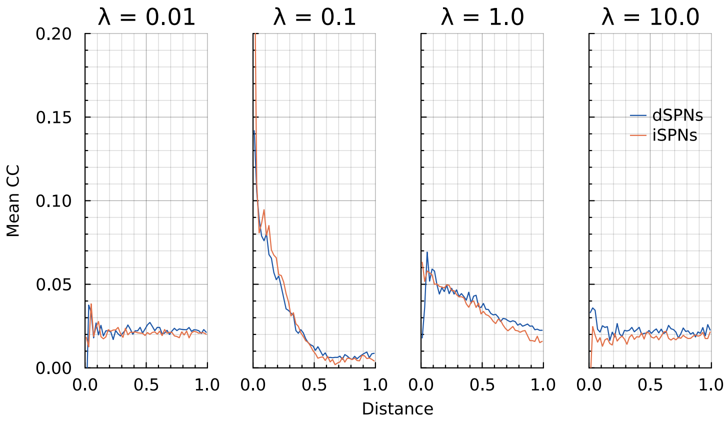

Fig. S2. Spatial correlations depends on dopamine spatial scale. Mean Pearson's correlation coefficient between the firing rates of pairs of SPNs in simulations with different dopamine spatial constant λ, as a function of the distance between them. The panel with $\lambda = 0.1$ shows the same data as Fig. 4F.

Table S1. Number of units per population in the model.

Population	Number of units
Cortex (exc)	160
Cortex (inh)	40
Thalamus	40
Striatum (dSPN)	100
Striatum (iSPN)	100
SNr/GPi	4
SNc	4

Table S2. Initial synaptic strength. Note that weights for inhibitory connections were multiplied by -1 after they are drawn.

pre	post	$w_{\max}$
Cortex (exc)	Cortex(exc)	25
Cortex (exc)	Cortex(inh)	25
Cortex (inh)	Cortex(exc)	25
Cortex (inh)	Cortex(inh)	25
Cortex (exc)	Striatum (dSPN)	25
Cortex (exc)	Striatum (iSPN)	25
Striatum (dSPN)	Striatum (dSPN)	25
Striatum (dSPN)	Striatum (iSPN)	25
Striatum (iSPN)	Striatum (dSPN)	25
Striatum (iSPN)	Striatum (iSPN)	25
Striatum (dSPN)	SNr/GPi	5
Striatum (iSPN)	SNr/GPi	5
Thalamus	Cortex (exc)	50
Thalamus	Cortex (inh)	50
Thalamus	Striatum (dSPN)	30
Thalamus	Striatum (iSPN)	30
